# Development and clinical application of a TaqMan-based real-time quantitative PCR assay for rapid detection of goose adenovirus type 4

**DOI:** 10.3389/fmicb.2026.1935172

**Published:** 2026-09-08

**Authors:** Rongchang Liu, Pan Tao, Dongqiang Hou, Long Zhao, Qizhang Liang, Weiwei Wang, Minhua Sun, Longfei Cheng, Chunhe Wan, Hongmei Chen, Nansong Jiang, Qiuling Fu, Guanghua Fu, Ming Liao, Yu Huang

**Affiliations:** 1 Fujian Provincial Key Laboratory for Avian Diseases Control and Prevention, Institute of Animal Husbandry and Veterinary Medicine, Fujian Academy of Agricultural Sciences, Fuzhou, China; 2 National and Regional Joint Engineering Laboratory for Medicament of Zoonosis Prevention and Control, Guangdong Provincial Key Laboratory of Zoonosis Prevention and Control, College of Veterinary Medicine, South China Agricultural University, Guangzhou, China; 3 Guangdong Engineering Technology Research Center of Biosafety and Intelligent Control for Aquatic Animals Diseases, Zhongkai University of Agriculture and Engineering, Guangzhou, China; 4 Guangdong Academy of Agricultural Sciences, Key Laboratory for prevention and control of Avian Influenza and Other Major Poultry Diseases, Ministry of Agriculture and Rural Affairs, Institute of Animal Health, Guangzhou, China

**Keywords:** goose adenovirus type 4, hexon gene, molecular diagnosis, TaqMan real-time quantitative PCR, waterfowl virology

## Abstract

**Introduction:**

Goose adenovirus type 4 (GoAdV-4) is an emerging pathogen that causes inclusion body hepatitis and hepatic necrosis in goslings, with mortality rates reaching up to 80% in severe outbreaks. Since its first identification in China in 2022, GoAdV-4 has spread rapidly across major goose-producing provinces, posing a serious threat to the domestic goose industry. However, no sensitive and specific quantitative assay has been available for the rapid diagnosis and viral load monitoring of GoAdV-4.

**Methods:**

We developed a TaqMan-based real-time quantitative PCR (qPCR) assay targeting the hexon gene of GoAdV-4 and systematically validated its analytical sensitivity, specificity, and repeatability. The assay was then applied to 582 clinical samples of five specimen types (tissues, blood, goose embryos, and cloacal swabs), and its diagnostic performance was compared with conventional PCR.

**Results:**

The assay demonstrated a linear detection range of 6.4 × 10^1^ to 6.4 × 10^7^ copies/μL (*R*^2^ = 0.9968) with an amplification efficiency of 97.5%, and a limit of detection (LOD) as low as 6.4 × 10^1^ copies/μL. No cross-reactivity was observed with fowl adenovirus serotype 4, duck adenovirus type 3, egg drop syndrome virus, goose circovirus, goose astrovirus, goose parvovirus, or nuclease-free water. Intra-assay and inter-assay coefficients of variation ranged from 0.87% to 1.17% and 1.06% to 1.48%, respectively, indicating high reproducibility. The assay identified 55 GoAdV-4-positive samples (9.45%), compared with 48 positives (8.25%) detected by conventional PCR, with an overall concordance rate of 98.80% (positive concordance 87.27%, negative concordance 100%).

**Discussion:**

The TaqMan qPCR assay detected seven additional positive samples missed by conventional PCR, all confirmed as true positives by Sanger sequencing, demonstrating superior sensitivity. These results show that the established TaqMan qPCR assay is a rapid, sensitive, specific, and reproducible tool for GoAdV-4 detection, providing a valuable diagnostic instrument for the surveillance and control of this emerging waterfowl pathogen.

## 1 Introduction

Adenoviruses are non-enveloped, icosahedral double-stranded DNA viruses approximately 70–100 nm in diameter that infect a wide range of vertebrate hosts spanning fish to mammals (Russell, 2009; Davison et al., 2003). According to the International Committee on Taxonomy of Viruses (ICTV), the family Adenoviridae currently comprises seven genera, of which the genus Aviadenovirus exclusively infects birds (Benko et al., 2022). Within this genus, 16 officially recognized species have been designated, including species with considerable veterinary importance in poultry and waterfowl, such as fowl aviadenovirus A–E, duck aviadenovirus A and B, and goose aviadenovirus A (McFerran and Smyth, 2000; Hess, 2000). Adenovirus infections in poultry can cause a range of diseases, including inclusion body hepatitis, hydropericardium hepatitis syndrome, and gizzard erosion, resulting in substantial economic losses worldwide (Adair and Fitzgerald, 2013).

Goose adenoviruses were first reported in by Csontos (1967), who isolated adenoviruses from goose flocks in Hungary. Subsequently, the virus spread throughout Hungary and neighboring regions, drawing increasing attention from the poultry industry. Zsák and Kisary (1984) isolated seven GoAdV strains from the livers and intestines of diseased 2- to 4-week-old goslings in Hungary and classified them into three genotypes (GoAdV-1, GoAdV-2, and GoAdV-3) based on restriction endonuclease cleavage patterns. Kaján et al., (2012) determined the complete genome sequence of the P29 strain, originally isolated from a dead gosling in the 1970s, and designated it goose adenovirus type 4 (GoAdV-4). The ICTV subsequently classified this strain within the species Goose aviadenovirus A, family Adenoviridae. The P29 genome is approximately 43.5 kb in length and contains 35 predicted protein-coding regions, with 19 genes derived from the common adenovirus ancestor, nine genes unique to aviadenoviruses, and seven genes exclusive to GoAdV-4, suggesting that this virus has evolved distinctive genomic features during its evolutionary history (Kaján et al., 2012).

In recent years, the rapid expansion of the waterfowl industry in China, coupled with increasing farming densities, has been accompanied by a growing threat from viral diseases (He et al., 2022). In April 2022, our research group isolated and identified the first GoAdV-4 strain outside Hungary from diseased goslings in Fujian Province, China (CH-FJZZ-202201 strain; GenBank accession number: OR842729) (Liu et al., 2024). Whole-genome sequencing revealed that the CH-FJZZ-202201 genome is 43,480 bp in length and shares 96.69% nucleotide identity with the Hungarian P29 strain, placing both strains within the same evolutionary branch (Liu et al., 2024). Epidemiological investigations indicated that GoAdV-4 infection causes morbidity and mortality rates of 10%−30% in infected goslings, although mortality can reach up to 80% in severe outbreaks under conditions of high stocking density or co-infection (Ivanics et al., 2010), with an expanding range of susceptible hosts and age groups. Li et al. (2025) isolated a novel GoAdV-4 strain (JA2485) from diseased Sanhua geese in Jiangxi Province, demonstrating 50% mortality in 1-day-old Sanhua geese via both subcutaneous injection and oral inoculation. These outbreaks indicate that GoAdV-4 has established a multi-focal presence in China and is undergoing continuous evolution. Notably, recent epidemiological surveys have revealed high prevalence rates of adenovirus infections in waterfowl across China, with frequent co-infections with other viral pathogens, underscoring the urgent need for reliable detection tools (Wu et al., 2024).

Despite the growing threat of GoAdV-4, laboratory detection methods remain limited. Traditional virus isolation requires 5–7 days and is technically demanding (Schachner et al., 2018). Electron microscopy requires expensive instrumentation. Serological methods are constrained by cross-reactivity and limited sensitivity. Therefore, rapid, sensitive, specific, and operationally simple molecular detection methods are urgently needed.

Real-time quantitative PCR (qPCR) (Thermo Fisher Scientific, Waltham, MA, USA) has become a widely adopted method for pathogen detection in veterinary diagnostics (Mackay et al., 2002). The TaqMan probe method employs a target-specific probe labeled with a fluorescent reporter and quencher. Fluorescence is generated only upon specific hybridization and cleavage during amplification, conferring higher specificity than dye-based methods through dual recognition (primers plus probe) (Heid et al., 1996).

The hexon protein is the most abundant adenovirus structural protein, containing both type-specific and group-specific determinants (Sohaimi and Hair-Bejo, 2021; Rux and Burnett, 2004). The hexon gene is highly conserved within the genus but harbors serotype-specific variable regions, making it an ideal target for specific detection (Crawford-Miksza and Schnurr, 1996; Pichla-Gullon et al., 2007). Hexon gene-based detection assays have been successfully established for several avian adenoviruses, supporting its utility as a diagnostic target. However, to our knowledge, no TaqMan-based qPCR assay specific for GoAdV-4 has been reported to date. A comprehensive literature search of PubMed, Web of Science, and CNKI using the keywords “goose adenovirus type 4, GoAdV-4, TaqMan and real-time PCR” did not identify any prior publication describing a TaqMan qPCR assay for this pathogen.

In this study, we designed TaqMan qPCR primers and a probe targeting the conserved region of the GoAdV-4 hexon gene, constructed a recombinant plasmid standard, optimized the reaction system, and systematically validated the assay. The established assay was subsequently applied to a large panel of 582 clinical samples representing five specimen types, and its diagnostic performance was evaluated against conventional PCR. This study addresses an unmet need for quantitative molecular detection of GoAdV-4.

## 2 Materials and methods

### 2.1 Virus strains and clinical samples

GoAdV-4 strain CH-FJZZ-202201 (GenBank: OR842729), fowl adenovirus serotype 4 (FAdV-4), duck adenovirus type 3 (DAdV-3), egg drop syndrome virus (EDSV), goose circovirus (GoCV), goose astrovirus (GoAstV), and goose parvovirus (GPV) were isolated, identified, and preserved by the Poultry Disease Laboratory, Institute of Animal Husbandry and Veterinary Medicine, Fujian Academy of Agricultural Sciences. A total of 582 clinical samples of five specimen types—tissues (liver, spleen, kidney; *n* = 212), Cloacal swabs (female geese and meat geese) (*n* = 146), blood (*n* = 124), goose embryos (*n* = 57), and Cloacal swabs (male breeding geese) (*n* = 43)—were collected from clinically suspected diseased geese across multiple provinces of China between 2021 and 2024.

### 2.2 Reagents and instruments

The PerfectStart^®^ II Probe qPCR SuperMix with UDG was purchased from TransGen Biotech (Beijing, China). The EasyPure Viral DNA/RNA Kit, pEASY-T1 Simple Cloning Kit, and 2 × EasyTaq PCR SuperMix were obtained from TransGen Biotech (Beijing, China). The pMD18-T vector and DL2000 DNA Marker were purchased from Takara Bio (Beijing, China). Gel extraction and plasmid mini-prep kits were obtained from Omega Bio-Tek (Norcross, GA, USA). The real-time fluorescence quantitative PCR instrument used was the ABI QuantStudio 3 (Thermo Fisher Scientific, Waltham, MA, USA). All primers and probes were synthesized by Sangon Biotech (Shanghai, China).

### 2.3 Primer and probe design

Based on the hexon gene sequences of GoAdV-4 strains CH-FJZZ-202201 (GenBank: OR842729) and P29 (GenBank: JF510462), nucleotide sequence alignment analysis was performed using Lasergene DNASTAR. Conserved regions were identified, and TaqMan qPCR primers and probe were designed using Oligo 7.0 software (Molecular Biology Insights, Inc., Cascade, CO, USA). The probe was labeled with FAM at the 5' end and BHQ1 at the 3' end. Specificity was verified by BLAST analysis. All primer and probe sequences are listed in Table 1. The predicted melting temperature (Tm) and GC content of each oligonucleotide, calculated using Oligo 7.0, are provided in Table 1. Secondary structure, self-dimer, and cross-dimer analyses performed with Oligo 7.0 showed no significant hairpin or dimer formation for any of the oligonucleotides.

**Table 1:** Primer and probe sequences used in this study.

Name	Sequence (5' → 3')	Tm (°C)	GC (%)	Fragment size (bp)
GoAdV-4-Hexon-F	TGTCTTTAGTAATGATGGGTAT	57.9	31.8	650
GoAdV-4-Hexon-R	CCTCATTAAACACTACGGAT	58.5	40.0	
GoAdV-4-Hexon-F1	TACCCGATAAATATAAAGTGAG	56.5	31.8	123
GoAdV-4-Hexon-R1	CTCCCACATTAGTAAATAGATC	57.4	36.4	
GoAdV-4-Hexon-P1	FAM-CTATATTGGTCAATGGCAC ACGCTT-BHQ1	61.2	40.0	

### 2.4 Nucleic acid extraction

Viral genomic DNA/RNA was extracted using the EasyPure Viral DNA/RNA Kit according to the manufacturer's instructions. For RNA viruses, the extracted RNA was reverse-transcribed to cDNA. All nucleic acids were stored at −80°C until use.

### 2.5 Construction of the recombinant plasmid standard

Using GoAdV-4 CH-FJZZ-202201 nucleic acid as template, the hexon gene fragment (650 bp) was amplified with primers GoAdV-4-Hexon-F/R. The PCR product was purified, cloned into pMD18-T vector, and transformed into *E. coli* DH5α. Positive clones were identified by blue-white screening and PCR. The recombinant plasmid (pMD18-Hexon) was quantified by spectrophotometry (235.7 ng/μL), corresponding to 6.4 × 10^10^ copies/μL, calculated using: copy number = [6.02 × 10^23^ × concentration (ng/μL) × 10^−9^] / molecular weight (g/mol). The plasmid was serially diluted to obtain standards from 6.4 × 10^9^ to 6.4 × 10^0^ copies/μL.

### 2.6 Establishment and optimization of the TaqMan qPCR assay

The TaqMan qPCR was performed in 20 μL containing 10 μL PerfectStart^®^ II Probe qPCR SuperMix (UDG), 0.4 μL each primer (10 μmol/L), TaqMan probe (10 μmol/L; final concentration optimized as described below), 2 μL template, and nuclease-free water. The probe concentration was optimized at 0.2, 0.4, 0.6, 0.8, and 1.0 μL, and annealing temperature at 56°C, 58°C, and 60°C. The optimized system: 10 μL SuperMix, 0.4 μL each primer, 0.4 μL probe, 2 μL template, water to 20 μL. Cycling: 25 °C 3 min; 95 °C 3 min; 40 cycles of 95 °C 10 s, 60 °C 30 s (fluorescence acquisition).

### 2.7 Standard curve construction

The plasmid standard was serially diluted 10-fold (6.4 × 10^3^ to 6.4 × 10^7^ copies/μL). Each dilution was tested in triplicate. Standard curves were generated by plotting Ct values against log_10_ of copy number. Linear regression equation, *R*^2^, and amplification efficiency {*E* = [10(^−1/slope) – 1] × 100%} were calculated. Acceptable efficiency: 90%−110% per MIQE guidelines (Bustin et al., 2009).

### 2.8 Analytical sensitivity

The plasmid standard was serially diluted 10-fold from 6.4 × 10^7^ to 6.4 × 10^0^ copies/μL (Eight levels). Each dilution was tested in triplicate. The limit of detection (LOD) was defined as the lowest concentration yielding reproducible positive amplification (Ct ≤ 34) across all replicates.

### 2.9 Specificity

Nucleic acids from GoAdV-4 and seven other waterfowl pathogens (FAdV-4, DAdV-3, EDSV, GoCV, GoAstV, GPV) and ddH_2_O were tested in triplicate. Specificity was assessed by presence or absence of amplification signals.

### 2.10 Repeatability

Four plasmid concentrations (6.4 × 10^2^ to 6.4 × 10^5^ copies/μL) were used for intra-assay (triplicate within one run) and inter-assay (three independent runs on different days, triplicate each) repeatability evaluation. Mean Ct, SD, and CV were calculated.

### 2.11 Clinical sample detection and method comparison

The 582 clinical samples described in Section 2.1 were tested in parallel by both TaqMan qPCR and conventional PCR (primers GoAdV-4-Hexon-F/R). Conventional PCR: 95 °C 5 min; 35 cycles of 95 °C 30 s, 55 °C 30 s, 72 °C 45 s; 72 °C 10 min. Products were analyzed by 1.5% agarose gel electrophoresis. Positive samples were confirmed by Sanger sequencing and BLAST. For qPCR, samples with Ct values ≤ 34 were considered positive, those with Ct values between 34 and 36 were considered suspicious and re-tested, and those with Ct values >36 or no Ct were considered negative. These Ct cutoff criteria were established based on the following: (1) the Ct values of all Sanger-sequencing-confirmed positive clinical samples in this study ranged from 15.38 to 34.21, with no confirmed positive sample exhibiting a Ct value >34; (2) the ≤ 34 threshold is consistent with the assay's analytical limit of detection (6.4 × 10^1^ copies/μL), which reproducibly yielded Ct values of approximately 32–34 across replicates; and (3) the 34–36 window was designated as a conservative borderline zone to account for stochastic amplification variability at near-LOD template concentrations — all samples falling within this range were confirmed by re-testing and Sanger sequencing. Discordant results between the two methods were confirmed by Sanger sequencing of the PCR product and BLAST analysis.

## 3 Results

### 3.1 Standard curve and amplification efficiency

**Figure 1:**
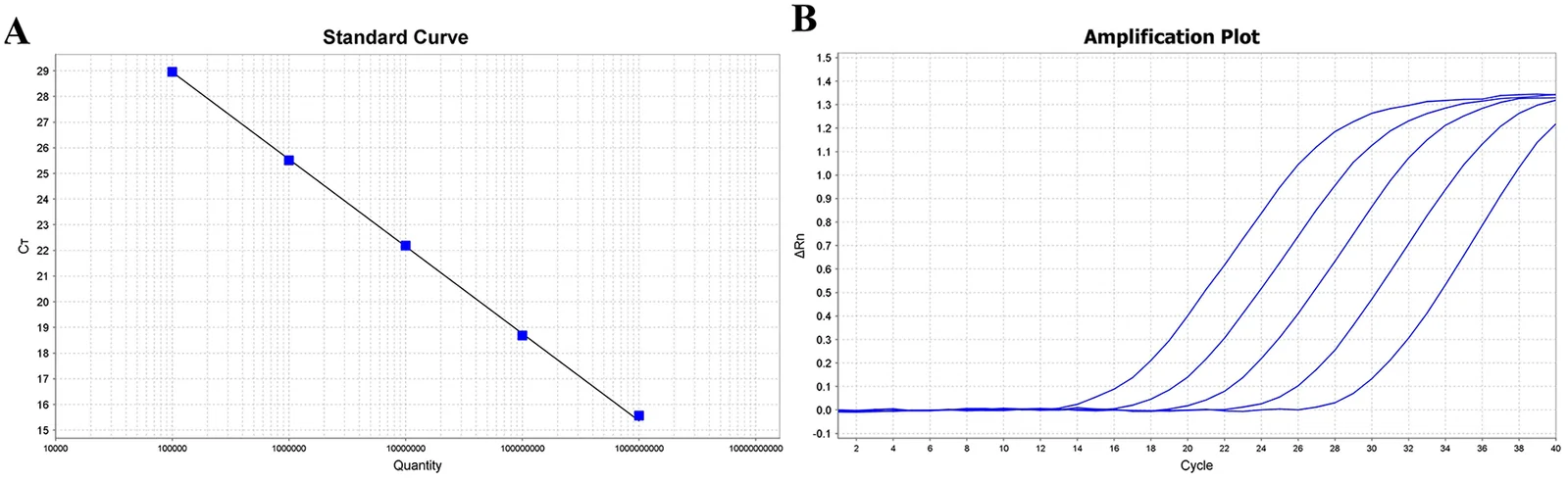
**(A)** Standard curve and **(B)** amplification curves of the TaqMan qPCR assay.

Five concentration gradients of the recombinant plasmid standard (6.4 × 10^3^ to 6.4 × 10^7^ copies/μL) were used for TaqMan qPCR. The amplification curves showed typical sigmoidal profiles with clear separation between adjacent dilution gradients (Figure 1B). The standard curve exhibited a strong linear relationship (Figure 1A). The linear regression equation was: y = −3.389x + 45.126, with *R*^2^ = 0.9968 and amplification efficiency *E* = 97.5%, within the ideal range of 90%−110% (Bustin et al., 2009). These results demonstrate excellent linearity, high amplification efficiency, and accurate quantification capability.

### 3.2 Analytical sensitivity

**Figure 2:**
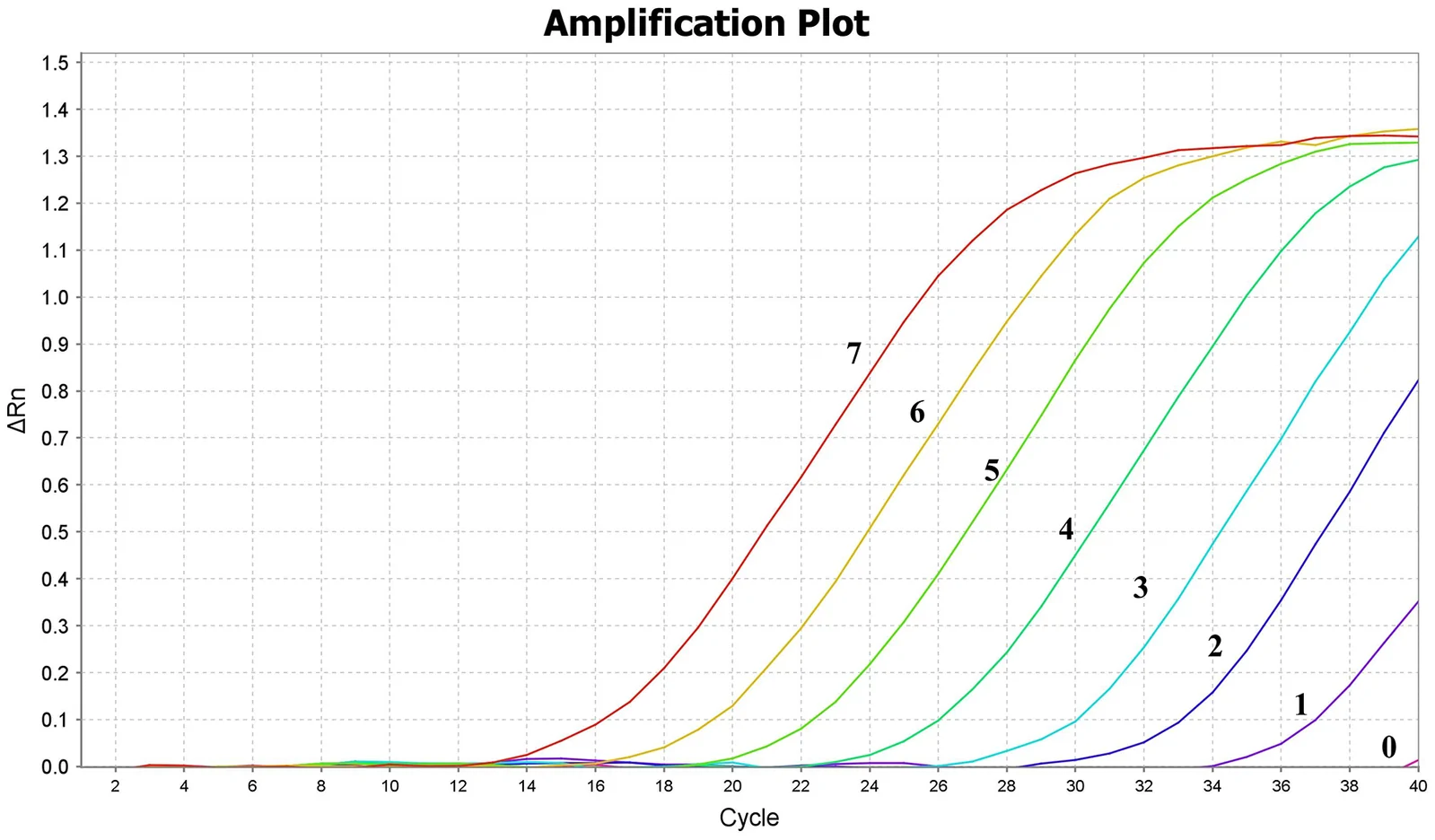
Sensitivity of the TaqMan qPCR assay. Amplification curves for 10-fold serial dilutions (6.4 × 10^7^ to 6.4 × 10^0^ copies/μL). LOD = 6.4 × 10^1^ copies/μL.

The analytical sensitivity was evaluated by testing 10-fold serial dilutions from 6.4 × 10^7^ to 6.4 × 10^0^ copies/μL. Specific amplification signals were consistently detected at concentrations as low as 6.4 × 10^1^ copies/μL (Ct < 34) across all three replicates (Figure 2). The LOD was therefore determined to be 6.4 × 10^1^ copies/μL.

### 3.3 Specificity

**Figure 3:**
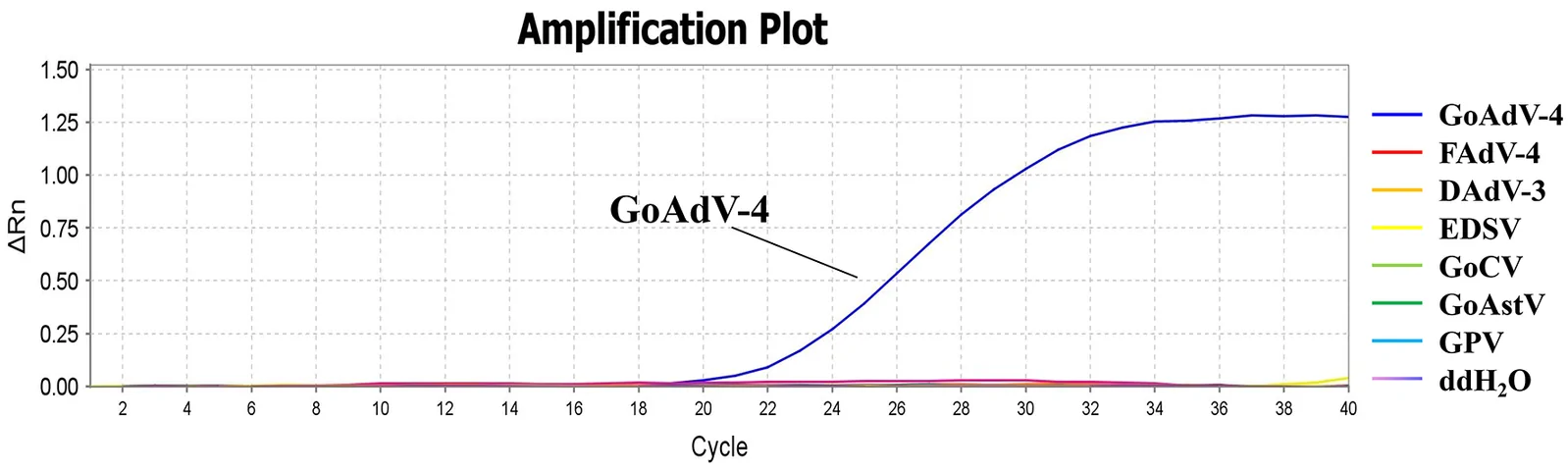
Specificity of the TaqMan qPCR assay.

Only GoAdV-4 produced a positive amplification signal. No amplification was detected for FAdV-4, DAdV-3, EDSV, GoCV, GoAstV, GPV, or ddH_2_O (Figure 3). This confirms that the TaqMan qPCR assay is highly specific for GoAdV-4, attributed to the dual-recognition mechanism requiring both primer binding and probe hybridization.

### 3.4 Repeatability

The repeatability results are shown in Table 2. Intra-assay CVs ranged from 0.87% to 1.17%, and inter-assay CVs ranged from 1.06% to 1.48% (Table 2). All values were well below the 5% threshold recommended by the MIQE guidelines (Bustin et al., 2009), indicating excellent repeatability and reproducibility.

**Table 2:** Intra-assay and inter-assay repeatability of the TaqMan qPCR assay.

Primer Name	Plasmid Concentration (copies/μL)	Intra-assay		Inter-assay	
		Mean (Ct)	CV (%)	Mean (Ct)	CV (%)
GoAdV-4-Hexon-P1	6.4 × 10^5^	18.696 ± 0.218	1.168	18.688 ± 0.276	1.476
	6.4 × 10^4^	22.022 ± 0.247	1.12	21.867 ± 0.275	1.257
	6.4 × 10^3^	25.83 ± 0.225	0.87	25.662 ± 0.321	1.252
	6.4 × 10^2^	29.872 ± 0.278	0.932	29.614 ± 0.313	1.056

### 3.5 Clinical sample detection

The TaqMan qPCR assay and conventional PCR were applied in parallel to 582 clinical samples encompassing five specimen types: tissues, blood, goose embryos, and Cloacal swabs (Table 3). By TaqMan qPCR, 55 samples (9.45%) tested positive for GoAdV-4. Conventional PCR detected 48 positive samples (8.25%). The detection rates by sample type are summarized in Table 3. Tissue samples showed the highest positivity rate by both qPCR (10.85%, 23/212) and conventional PCR (10.38%, 22/212), followed by Cloacal swabs (female geese and meat geese) (qPCR: 10.27%, 15/146; PCR: 8.90%, 13/146), blood (qPCR: 9.68%, 12/124; PCR: 8.87%, 11/124), and goose embryos (qPCR: 8.77%, 5/57; PCR: 3.51%, 2/57). No positive samples were detected in Cloacal swabs (male breeding geese) (*n* = 43) by either method. The concordance between the two methods is shown in Table 4. Among the 55 qPCR-positive samples, 48 were also positive by conventional PCR, while seven were PCR-negative, yielding a positive concordance (sensitivity) of 87.27% (48/55). All 527 qPCR-negative samples were also PCR-negative, resulting in a negative concordance (specificity) of 100% (527/527). The overall concordance rate was 98.80% [(48 + 527) / 582]. Sanger sequencing and BLAST analysis confirmed that all seven discordant samples (qPCR-positive/PCR-negative) were true GoAdV-4 positives. These results demonstrate that the TaqMan qPCR assay offers superior sensitivity compared with conventional PCR, while maintaining complete specificity.

**Table 3:** Detection of GoAdV-4 in different clinical specimen types by TaqMan qPCR and conventional PCR.

Specimen type	No. of samples	PCR positive	PCR positive rate (%)	qPCR positive	qPCR positive rate (%)
Tissue	212	22	10.38	23	10.85
Cloacal swabs (female geese and meat geese)	146	13	8.90	15	10.27
Blood	124	11	8.87	12	9.68
Goose embryo	57	2	3.51	5	8.77
Cloacal swabs (male breeding geese)	43	0	0	0	0
Total	582	48	8.25	55	9.45

qPCR positivity criteria: Ct ≤ 34, positive; 34 < Ct ≤ 36, suspicious (confirmed by re-testing); Ct >36 or no Ct, negative [The Ct thresholds were determined based on the Ct distribution of sequencing-confirmed positive clinical samples (all ≤ 34.21) and the assay's validated analytical limit of detection (6.4 × 10^2^ copies/μL, corresponding to Ct ≈ 32–34)].

**Table 4:** Concordance between TaqMan qPCR and conventional PCR for GoAdV-4 detection in clinical samples.

qPCR result	PCR positive	PCR negative	Total
qPCR positive	48	7	55
qPCR negative	0	527	527
Total	48	534	582

Overall concordance rate: 98.80% [(48 + 527) / 582]; Positive concordance (sensitivity): 87.27% [48 / (48 + 7)]; Negative concordance (specificity): 100% [527 / (527 + 0)].

## 4 Discussion

This study presents a TaqMan-based qPCR assay specifically targeting GoAdV-4 that has been systematically validated for clinical use. Since its first identification in Hungary in 1967 (Csontos, 1967), GoAdV-4 remained geographically restricted for over five decades. However, the 2022 isolation in Fujian Province, China (Liu et al., 2024) and subsequent reports from Jiangxi Province (Li et al., 2025) confirmed the transcontinental spread of this virus. The high mortality rates (10%−30%) and expanding host range underscores the urgency of developing reliable diagnostic tools. The growing recognition of adenovirus-associated diseases in poultry worldwide has further driven demand for improved molecular diagnostic capabilities (Chen, 2015; Li et al., 2017). Furthermore, severe adenovirus-associated hepatitis and hydropericardium have been documented in goslings in Hungary, confirming the broader pathogenic potential of goose adenoviruses (Ivanics et al., 2010).

The selection of the hexon gene as the detection target was critical to the assay's performance. As the predominant adenovirus capsid component, the hexon protein is encoded by a single-copy gene that contains both genus-level conserved regions and serotype-specific variable regions (Crawford-Miksza and Schnurr, 1996; Pichla-Gullon et al., 2007). Our comprehensive sequence alignment of GoAdV-4 hexon gene sequences from multiple strains, including CH-FJZZ-202201 and P29, ensured both broad coverage of circulating variants and high specificity against non-target avian viruses.

The established assay demonstrated excellent performance. The standard curve *R*^2^ of 0.9968 and efficiency of 97.5% exceed the MIQE guideline requirements (Bustin et al., 2009). It should be noted that the standard curve was constructed using plasmid DNA (pMD18-Hexon) rather than genomic viral DNA. While plasmid-based standards are widely used in qPCR assay development and offer advantages in stability and precise quantification, differences in supercoiling, molecular size, and secondary structure may introduce minor systematic bias in absolute copy number estimation compared with genomic DNA. In our study, the clinical concordance between TaqMan qPCR and conventional PCR (98.80%) and the confirmation of all discordant positives by Sanger sequencing suggest that any such matrix effect, if present, did not meaningfully impact diagnostic accuracy. The LOD of 6.4 × 10^1^ copies/μL is comparable to or better than those reported for other avian adenovirus qPCR assays, enabling the identification of early-stage or low-level infections. The complete specificity against seven non-target pathogens confirms the dual-recognition advantage of the TaqMan format over SYBR Green I methods, which require post-amplification melt curve analysis and are more susceptible to false-positive signals from non-specific amplification (Ponchel et al., 2003). The low intra- and inter-assay CVs (all ≤ 1.48%; Table 2) further confirm the robustness and reliability of the assay.

The TaqMan qPCR assay offers distinct advantages over existing approaches. Compared with virus isolation, which requires 5–7 days (Schachner et al., 2018), results are available within 2 h. Compared with SYBR Green I qPCR, the dual-recognition mechanism eliminates the need for melt curve analysis and reduces false-positive risk (Heid et al., 1996). The quantitative capability enables viral load monitoring, providing information that conventional PCR cannot offer. Multiplex PCR approaches (Chen et al., 2013; Song et al., 2025) could complement this method for comprehensive pathogen screening in future studies.

Clinical application to 582 samples across five specimen types revealed a 9.45% GoAdV-4 positivity rate by TaqMan qPCR (Table 3), confirming broad geographic distribution across Fujian, Guangdong, Jiangxi, Zhejiang, Anhui, Jiangsu, and other major goose-producing provinces. The positive samples encompassed diverse sample types including tissues, Cloacal swabs, blood, and goose embryos, indicating multiple routes of virus shedding and transmission. Notably, the TaqMan qPCR assay detected seven additional positive samples that were missed by conventional PCR (Table 4), all confirmed as true positives by Sanger sequencing, demonstrating the superior sensitivity of the qPCR assay. The 100% negative concordance (specificity) further confirms the assay's exceptional clinical reliability. Tissue samples exhibited the highest positivity rate (10.85%; Table 3), supporting their utility as the preferred specimen type for GoAdV-4 molecular diagnosis. The quantitative capability of the assay further enables viral load assessment, providing valuable data for epidemiological surveillance and disease severity evaluation.

Despite the robust sample size, several limitations should be acknowledged. First, validation with additional specimen types (e.g., feces, environmental samples) would further broaden the assay's applicability. Second, the potential for hexon gene mutations to affect primer or probe binding over time warrants ongoing sequence surveillance. Notably, the primer and probe binding sites were located within conserved regions identified by alignment of the hexon gene sequences of strain CH-FJZZ-202201 (isolated in Fujian, China, in 2022) and strain P29 (isolated in Hungary in the 1970s), which share 96.69% overall nucleotide identity (Liu et al., 2024; Kaján et al., 2012) — suggesting that these target sites are evolutionarily stable across geographically and temporally distant GoAdV-4 strains. Nevertheless, given the ongoing evolution of GoAdV-4, we recommend periodic re-sequencing of the hexon gene from circulating field isolates and BLAST verification of primer/probe complementarity as part of routine assay quality assurance. Third, the current sample collection may be subject to regional sampling bias. Future research should focus on: (1) large-scale epidemiological surveys across China; (2) monitoring hexon gene sequence variations; (3) developing multiplex TaqMan qPCR for simultaneous detection of GoAdV-4 and other goose pathogens; (4) investigating viral load–disease severity correlations; and (5) standardizing the assay into a commercial kit.

## 5 Conclusion

A TaqMan-based real-time quantitative PCR assay targeting the hexon gene was successfully developed for the rapid, sensitive, specific, and reproducible detection of GoAdV-4. The assay demonstrated a LOD of 6.4 × 10^1^ copies/μL, complete specificity against seven common waterfowl pathogens, and excellent repeatability (all CVs < 1.48%). Clinical application to 582 clinical samples of five specimen types revealed a 9.45% positivity rate by qPCR, with seven additional positive samples detected compared with conventional PCR, and an overall concordance of 98.80%. This study provides a much-needed addition to the molecular diagnostic toolkit for GoAdV-4 and provides a reliable platform for early diagnosis, large-scale epidemiological surveillance, and quantitative viral load monitoring in both clinical and field settings.

## Data Availability

The original contributions presented in the study are included in the article/supplementary material, further inquiries can be directed to the corresponding authors.

## References

[B1] Adair B. M. Fitzgerald S. D. (2013). “Adenoviruses,” in Diseases of Poultry, 13th Edn, ed. D. E. *Swayne* (Ames, IA: Wiley-Blackwell), 322–342.

[B2] Benko M. Aoki K. Arnberg N. Davison A. J. Derypere F. Gultyaev A. P. . (2022). ICTV Virus Taxonomy Profile: Adenoviridae 2022. *J. Gen*. Virol. 103, 001721. doi: 10.1099/jgv.0.001721 35262477 PMC9176265

[B3] Bustin S. A. Benes V. Garson J. A. Hellemans A. Huggett J. Kubista M. . (2009). The MIQE guidelines: minimum information for publication of quantitative real-time PCR experiments. Clin. Chem. 55, 611–622. doi: 10.1373/clinchem.2008.112797 19246619

[B4] Chen P. Y. (2015). Veterinary Infectious Diseases, 6th Edn. Beijing: China Agricultural Press.

[B5] Chen Z. Y. Li C. F. Zhu Y. Q. (2013). Rapid diagnosis of goose viral infections by multiplex PCR. J. Virol. Methods 189, 53–57. doi: 10.1016/j.jviromet.2012.09.027 23518397

[B6] Crawford-Miksza L. Schnurr D. P. (1996). Analysis of 15 adenovirus hexon proteins reveals the location and structure of seven hypervariable regions containing serotype-specific residues. J. Virol. 70, 1836–1844. doi: 10.1128/jvi.70.3.1836-1844.1996 8627708 PMC190011

[B7] Csontos L. (1967). Isolation of adenoviruses from geese: preliminary report. *Acta Vet. Acad. Sci*. Hung. 17, 217–219. 6070936

[B8] Davison A. J. Benko M. Harrach B. (2003). Genetic content and evolution of adenoviruses. *J. Gen*. Virol. 84, 2895–2908. doi: 10.1099/vir.0.19497-0 14573794

[B9] He D. Wang F. Zhao L. Jiang X. Zhang S. Wei F. . (2022). Epidemiological investigation of infectious diseases in geese on mainland China during 2018–2021. Transbound. Emerg. Dis. 69, 3419–3432. doi: 10.1111/tbed.14699 36088652

[B10] Heid C. A. Stevens J. Livak K. J. Williams P. M. (1996). A novel method for real time quantitative RT-PCR. Genome Res. 6, 986–994. doi: 10.1101/gr.6.10.986 8908518

[B11] Hess M. (2000). Detection and differentiation of avian adenoviruses: a review. Avian Pathol. 29, 195–206. doi: 10.1080/03079450050045440 19184805

[B12] Ivanics É. Palya V. Markos B. Glávits R. Ivanics É. (2010). Hepatitis and hydropericardium syndrome associated with adenovirus infection in goslings. Acta Vet. Hung. 58, 47–58. doi: 10.1556/avet.58.2010.1.5 20159738

[B13] Kaján G. L. Davison A. J. Palya V. Ivanics É. Glávits R. Zádori Z. . (2012). Genome sequence of a waterfowl aviadenovirus, goose adenovirus 4. *J. Gen*. Virol. 93, 2457–2465. doi: 10.1099/vir.0.042028-0 22894926

[B14] Li B. J. Chang J. J. Cao X. Y. Zhou W. W. Liu L. Gao W. M. . (2025). Emergence and pathogenicity of a novel goose adenovirus type 4 strain JA2485: insights for poultry disease control. Viruses 17, 1560. doi: 10.3390/v17121560 PMC12737765 41472231

[B15] Li P. H. Zheng P. P. Zhang T. F. Wen G. Y. Shao H. B. Luo Q. P. (2017). Fowl adenovirus serotype 4: epidemiology, pathogenesis, diagnostic detection, and vaccine strategies. Poult. Sci. 96, 2630–2640. doi: 10.3382/ps/pex087 28498980

[B16] Liu R. Sun M. Lan Q. Zhang J. Liang Q. Fu G. . (2024). First report of a novel goose adenovirus outbreak in Lion Head geese in China. Transbound. Emerg. Dis. 2024:3980468. doi: 10.1155/2024/3980468 PMC12017204 40303045

[B17] Mackay I. M. Arden K. E. Nitsche A. (2002). Real-time PCR in virology. Nucleic Acids Res. 30, 1292–1305. doi: 10.1093/nar/30.6.1292 PMC101343 11884626

[B18] McFerran J. B. Smyth J. A. (2000). Avian adenoviruses. Rev. Sci. Tech. 19, 589–601. doi: 10.20506/rst.19.2.1238 10935281

[B19] Pichla-Gullon S. L. Drinker M. Zhou X. Xue F. Rux J. J. Gao G. P. . (2007). Structure-based identification of a major neutralizing site in an adenovirus hexon. J. Virol. 81, 1680–1689. doi: 10.1128/JVI.02023-06 17108028 PMC1797575

[B20] Ponchel F. Toomes C. Bransfield K. Leong F. Douglas S. H. Field S. L. . (2003). Real-time PCR based on SYBR-Green I fluorescence: an alternative to the TaqMan assay for a relative quantification of gene rearrangements, gene amplifications and micro gene deletions. BMC Biotechnol. 3:18. doi: 10.1186/1472-6750-3-18 PMC270040 14552656

[B21] Russell W. C. (2009). Adenoviruses: update on structure and function. *J. Gen*. Virol. 90, 1–20. doi: 10.1099/vir.0.003087-0 19088268

[B22] Rux J. J. Burnett R. M. (2004). Adenovirus structure. Hum. Gene Ther. 15, 1167–1176. doi: 10.1089/hum.2004.15.1167 15684694

[B23] Schachner A. Matos M. Grafl B. (2018). Fowl adenovirus-induced diseases and strategies for their control: a review on the current global situation. Avian Pathol. 47, 111–126. doi: 10.1080/03079457.2017.1385724 28950714

[B24] Sohaimi N. M. Hair-Bejo M. (2021). A recent perspective on fiber and hexon genes proteins analyses of fowl adenovirus toward virus infectivity: a review. *Open Vet*. J. 11, 569–580. doi: 10.5455/OVJ.2021.v11.i4.6 PMC8770197 35070851

[B25] Song H. Kim H. Kim J. Her M. Kim H. (2025). A novel species-specific multiplex PCR assay for the differentiation of five Fowl adenovirus species (A to E): application to field surveillance. *Poult*. Sci. 104, 105856. doi: 10.1016/j.psj.2025.105856 41016164 PMC12510196

[B26] Wu B. Jiang X. He D. Wei F. Mao M. Zhu Y. . (2024). Epidemiological investigation of fowl adenovirus (FAdV) infections in ducks and geese in Shandong Province, China. Avian Pathol. 53, 155–163. doi: 10.1080/03079457.2024.2302138 38206316

[B27] Zsák L. Kisary J. (1984). Characterisation of adenoviruses isolated from geese. Avian Pathol. 13, 239–252. doi: 10.1080/03079458408418529 18766842

